# Single cell transcriptional diversity and intercellular crosstalk of human liver cancer

**DOI:** 10.1038/s41419-022-04689-w

**Published:** 2022-03-24

**Authors:** Yan Meng, Yan Sang, Jianping Liao, Qiudong Zhao, Shuping Qu, Rong Li, Jinghua Jiang, Meifeng Wang, Jiahong Wang, Dong Wu, Chun Cheng, Lixin Wei

**Affiliations:** 1 School of Medicine & Holistic Integrative Medicine, Nanjing University of Chinese Medicine, 138 Xianlin Street, Nanjing, 210023 China; 2 Tumor Immunology and Gene Therapy Center, Third Affiliated Hospital of Second Military Medical University, 225 Changhai Road, Shanghai, 200438 China; 3 Nursing Department, Affiliated Hospital of Nantong University, Nantong University, Nantong, 226001 China; 4 The School of Basic Medical Sciences of Fujian Medical University, Fujian Medical University, Fuzhou, 350108 China; 5 Department of Hepatic Surgery, Third Affiliated Hospital of Second Military Medical University, 225 Changhai Road, Shanghai, 200438 China

**Keywords:** Tumour heterogeneity, Cancer stem cells

## Abstract

Liver cancer arises from the evolutionary selection of the dynamic tumor microenvironment (TME), in which the tumor cell generally becomes more heterogeneous; however, the mechanisms of TME-mediated transcriptional diversity of liver cancer remain unclear. Here, we assess transcriptional diversity in 15 liver cancer patients by single-cell transcriptome analysis and observe transcriptional diversity of tumor cells is associated with stemness in liver cancer patients. Tumor-associated fibroblast (TAF), as a potential driving force behind the heterogeneity in tumor cells within and between tumors, was predicted to interact with high heterogeneous tumor cells via COL1A1-ITGA2. Moreover, COL1A1-mediated YAP-signaling activation might be the mechanistic link between TAF and tumor cells with increased transcriptional diversity. Strikingly, the levels of COL1A1, ITGA2, and YAP are associated with morphological heterogeneity and poor overall survival of liver cancer patients. Beyond providing a potential mechanistic link between the TME and heterogeneous tumor cells, this study establishes that collagen-stimulated YAP activation is associates with transcriptional diversity in tumor cells by upregulating stemness, providing a theoretical basis for individualized treatment targets.

## Introduction

Most liver cancer cases develop mainly due to evolutionary selection of an adverse tumor microenvironment (TME), in which deterministic tumor features are preferentially selected for their survival fitness [1, 2]. Accordingly, complex genomics and TMEs create a molecular conundrum for the diagnosis and treatment of liver cancer and contribute to therapeutic failure and ultimately lethal outcomes [2]. Tumor cells with strong stemness can generate heterogeneous subtypes through multidirectional differentiation [3, 4]. However, the underlying mechanisms of TME-mediated heterogeneity in liver cancer remain unclear. Molecular characterization of cell communities at the single-cell level may help shed light on the complex interplay among tumor cells and stromal cells in the TME.

Liver cancer is etiologically and biologically heterogeneous, comprising many molecular subtypes [5, 6], which are clinically treated as separate entities. Interestingly, there is evidence that some molecular subtypes of hepatocellular carcinoma (HCC) and intrahepatic cholangiocarcinoma (iCCA) share similar tumor biology and key drivers [7]. Accordingly, the hepatic microenvironment could direct lineage commitment to either HCC or iCCA in the presence of the same oncogenic drivers [8, 9]. The TME likely improves the capability of tumor cells to grow in poor microenvironment [2, 8, 9]. The cellular components of the TME are highly complex resulting in high microenvironmental diversity associated with poor prognosis [2, 9]. As an important constituent of the TME, tumor-associated fibroblast (TAF) is regarded as a promising therapeutic target for limiting cancer progression [10]. Studies have established that TAF could facilitate cancer progression via aberrant extracellular matrix (ECM) remodeling with collagen I (COL-I) enrichment [10, 11]. However, whether and how TAF regulates the heterogeneity of tumor cells is unclear, and requires further investigation.

The Hippo-Yes-associated protein (YAP) signaling is known to regulate stem cell homeostasis, tissue regeneration and tumor progression [12]. YAP is a major downstream effector of Hippo pathway [12, 13]. Phosphorylation of YAP results in its cytoplasmic retention and degradation [14, 15]. When dephosphorylated, YAP can enter the nucleus and bind with transcriptional factors to regulate the expression of many target genes [16], usually increasing cell proliferation and decreasing apoptosis [17]. YAP hyperactivation has been observed in many tumors, including liver cancer [13, 18]. However, whether and how Hippo-YAP signaling responds to TME stimuli to affect the tumor heterogeneity of liver cancer remains unknown.

Here, upon assessing the single-cell sequencing results of liver cancer patients, we identified transcriptional diversity of tumor cells potentially coexisted with the increased stemness. TAF interacted with high heterogeneous tumor cells via COL1A1-ITGA2, which activated YAP-signaling to regulate transcriptional diversity in tumor cells by improving their stemness. Thus, our study uncovered intercellular crosstalk between TAF and tumor cells involved in transcriptional diversity in liver cancer, suggesting potential targets for liver cancer therapy.

## Experimental procedures

### Cell culture and establishment of primary tumor-associated fibroblasts (TAFs)

HCC cell lines (Huh7, LM-3, and HepG2) were purchased from ATCC and cultured in DMEM media (Gibco) with Fetal Bovine Serum (FBS, 10%; Gibco) and Penicillin-Streptomycin (100 U; Gibco) at 37 °C, 5% CO2. Fresh human tumor tissues were used to harvest primary TAFs. Tissue samples were cut into small pieces (approximately 3 mm^3^) and put on to six-well cell culture plates with Dulbecco’s Modified Eagle’s Medium (DMEM) containing 10% FBS (Gibco), 10 ng/mL basic fibroblast growth factor (bFGF), 100 U/mL penicillin and 100 mg/mL streptomycin (Gibco). TAFs were starting to migrate out from the small piece of tissues from 3 to 7 days later. After 2 weeks, the remnants of the tissue were carefully removed and subcultured TAFs every 3 days, and passages 3–10 were used for this study [19, 20].

### Reagents and antibodies

DEN (N0756) and antibodies specific for Flag (M3165) were obtained from Sigma-Aldrich (MO). Antibodies specific for YAP (sc-101199, immunofluorescence [IF]) were purchased from Santa Cruz Biotechnology (TX), and those specific for YAP (ab39361, IP/IHC), ITGA2 (ab181548), and COL1A1 (ab34710) were purchased from Abcam (MA).

### Animal experiments

Male SD rats (10–12 weeks, 220–250 g) were obtained from the Shanghai Experimental Center, Chinese Science Academy, Shanghai. The rats were randomly grouped and maintained at an animal facility under pathogen-free conditions. All animal experiments were performed according to the animal protocols approved by the Shanghai Eastern Hepatobiliary Surgery Hospital Animal Care Committee. To induce the model of liver cancer, 100 p.p.m. DEN (95 μg/ml) was added to the drinking water of rats for 16 weeks. Liver tumors were measured with electronic calipers and counted (for tumors with diameters ≥1 mm). Liver sections were preserved in 10% neutral-buffered formalin for histopathological and immunohistochemistry (IHC) analyses, blood was collected, and serum was isolated for biochemical analysis. All analysis were conducted in investigator blinded fashion.

### Sphere formation assay

Sphere formation assays were performed in 6-cm culture dishes coated with 1% agarose. HPCs suspended in serum-free medium were seeded at a density of 5 000 cells/dish and incubated for 3–7 days in low or high concentrations of collagen. The numbers and sizes of spheres were observed manually under a microscope.

### Tissue microarray and IHC staining

Tissue microarray (TMA) sections of tumor and adjacent nontumor specimens were prepared by Shanghai Outdo Biotech Co., Ltd. (Shanghai, China). This TMA contains tissues from 77 paired fresh liver carcinoma and adjacent tumor tissue samples, and was used to examine the expression profiles of YAP, ITGA2 and COL1A1 by IHC. For IHC, TMA sections were incubated with anti-ITGA2 antibody (1:200 dilution), anti-YAP1 antibody (1:100 dilution), or anti-COL1A1 antibody (1:200 dilution). IHC staining was scored by two independent pathologists who were blinded to the clinical characteristics of the patients. The scoring system was based on the intensity and extent of staining: staining intensity was classified as 0 (negative), 1 (weak), 2 (moderate), or 3 (strong); and the staining extent was dependent on the percentage of positive cells (out of 200 examined cells) and was classified as 0 (<5%), 1 (5–25%), 2 (26–50%), 3 (51–75%), or 4 (>75%). According to the staining intensity and staining extent scores, the IHC results were classified as 0–1, negative (-); 2–4, weakly positive (+); 5–8, moderately positive (++), and 9–12, strongly positive (+++).

### Immunofluorescent staining

For antigen colocalization studies, fluorescence immunostaining of multiple proteins in tissues was performed with a sequential fluorescent method. Primary antibodies of against ITGA2 (1:200 dilution), YAP (1:100 dilution) and COL1A1 (1:200 dilution) were used. Alexa 488-conjugated goat antimouse IgG (Invitrogen, Carlsbad, CA) Alexa 561-conjugated goat antirabbit IgG (Invitrogen) and Alexa 647-conjugated goat antirabbit IgG were used as secondary antibodies.

### Real-time quantitative polymerase chain reaction (RT-PCR)

Total RNA was extracted from cells by using TRIzol Reagent (Invitrogen, Carlsbad, CA, USA), and further treated with RNase free DNase (Promega, Madison, WI, USA) to eliminate any residual DNA. Complementary DNA was prepared by using oligo dT18-primers and MMLV reverse transcriptase (Promega). RT-PCR was performed on a Light Cycler 480 system (Roche Diagnostics, Mannheim, Germany). The primers used in this experiment were as follows: *SMAD2*, forward, 5′-CCCACTCCATTCCAGAAAAC-3′, and reverse, 5′-GAGCCTGTGTCCATACTTTG-3′; *SOX9*, forward, 5′-AGGAAGCTGGCAGACCAGTA-3′, and reverse, 5′-ACGAAGGGTCTCTTCTCGCT-3′; *MYC*, forward, 5′-AACAGGAACTATGACCTCG-3′, and reverse, 5′-AGCAGCTCGAA TTTCTTC-3′; and *FGF8*, forward, 5′-CAGTTGGAATTGTGGCAATCAAAG-3′, and reverse, 5′-CTTTTGATTTAAGGCAACGAACATTTC-3′.

### Patients and follow-up analysis

The cohort in this study contained 77 patients from January 1997 to December 2007. All patients were randomly selected from those with liver cancer who underwent hepatectomy in the Shanghai Eastern Hepatobiliary Surgery Hospital. Informed consent was obtained from each patient under a protocol approved by the Hospital Research Ethics Committees. None of the patients were administered preoperative treatment, and recurrence was confirmed by contrast-enhanced imaging studies or cholangiography according to standard guidelines for liver cancer. Overall survival (OS) was defined as the interval between surgery and either death or the last follow-up. The data were censored at the last follow-up for surviving patients.

### Transfection and viral Infection

Approximately 0.5–2 µg/ml plasmid was transfected using Lipofectamine 2000 (Invitrogen) according to the manufacturer’s instructions. Where indicated, cells were infected with virus expressing GFP (multiplicity of infection (MOI), 10) or YAP (MOI, 10) in serum-free medium for the indicated times. Eight hours later, the cells were rinsed and cultured in fresh medium. After 48 h, the cells were cultured in DMEM supplemented with 10% FBS.

### ChIP-qPCR

ChIP was performed as previously described [21]. Briefly, cells were cross-linked with freshly prepared formaldehyde (1.42%) for 15 min, and treated with glycine (125 mM) for 5 min at room temperature. After two rounds of washing with ice-cold PBS, the cells were scraped and collected by centrifugation. Pelleted cells were resuspended in 400 μL of ChIP lysis buffer (50 mM HEPES/KOH, pH 7.5; 140 mM NaCl; 1 mM EDTA; 1% Triton X-100; 0.1% Na-deoxycholate and protease inhibitors) and subjected to sonication with Bioruptor to shear the chromatin (30 s on high-power, 30 s off; 20 cycles). After sonication, samples were further diluted twice with lysis buffer and centrifuged to clear the supernatant. Eighty microliters of supernatant (1/10 of total) were directly processed to extract total DNA as whole-cell input. The remaining supernatants were transferred to new Eppendorf tubes and incubated with either IgG or YAP antibodies (14074, Cell Signaling Technology) at 4 °C overnight. Samples were then treated with prewashed protein A/G beads (L2118; Santa Cruz) for another 3 h, washed five times with the indicated buffers and resuspended in 100 μL of 10% Chelex (1421253; Bio-Rad). The samples were boiled for 10 min and centrifuged at 4 °C for 1 min. Supernatants were transferred to new tubes. After that, another 120 μL of Milli-Q water was added to each bead pellet, which was vortexed for 10 s, and centrifuged again to spin down the beads. The supernatants were combined together as templates for follow-up qPCR analysis.

### ChIP-seq

ChIP-seq was performed based on a previous protocol with minor modifications [22]. Cells stably expressing Flag-tagged YAP were subjected to the same treatments as described above to obtain cell pellets. After that, cells were resuspended in 400 μL ChIP digestion buffer (20 mM Tris HCl, pH 7.5; 15 mM NaCl; 60 mM KCl; 1 mM CaCl2 and protease inhibitors). To shear the chromatin, cells were digested with an appropriate amount of micrococcal nuclease (MNase, M0247S, NEB) at 37 °C for 20 min to ensure that the majority of chromatin was mono- and di-nucleosomes. The reaction was stopped with 2X stop buffer (100 mM Tris HCl, pH 8.1; 20 mM EDTA; 200 mM NaCl; 2% Triton X-100; 0.2% Na-deoxycholate and protease inhibitors). Samples were further sonicated with a Bioruptor at high power for 15 cycles (30 s on, 30 s off) and then centrifuged to remove debris. Next, soluble chromatin was immunoprecipitated with FLAG antibodies (F3165, Sigma) at 4 °C together with prewashed protein A/G beads. After extensive washing with the indicated buffers, samples were eluted and reverse cross-linked in elution buffer (10 mM Tris HCl, pH 8.0; 10 mM EDTA; 150 mM NaCl; 5 mM DTT and 1% SDS) at 65 °C overnight. Then, sequential digestion with DNase and Proteinase K was performed, and the DNA was purified with a PCR purification kit (B518141, Sangon Biotech). DNA that was successfully collected from three ChIP assays was pooled to generate libraries with the Ovation Ultra-Low Library Prep kit (NuGEN) according to the manufacturer’s instructions. Sequencing was performed on an Illumina HiSeq 2500 platform.

### Cell dissociation

HCC tumor tissues and adjacent tissues were collected after surgical resection in MACS Tissue Storage Solution (Miltenyi Biotec) in a 50 mL conical tube and transported on ice to the laboratory. Briefly, samples were first washed with phosphate-buffered saline (PBS), minced into small pieces (approximately 1mm^3^) on ice, and enzymatically digested with collagenase I (Worthington) for 15 min at 37 °C, with agitation. After digestion, samples were sieved through a 70 µm cell strainer, and centrifuged at 300 g for 5 min. The cell pellet was resuspended in 1 mL freezing media (Gibco) for long-term cryopreservation in liquid nitrogen. Throughout the dissociation procedure, cells were maintained on ice whenever possible, and the entire procedure was completed in less than 1 hr.

### scRNA-seq data processing

ScRNA-seq data of tumor samples (*n* = 15; consistent with the original article, the sample would be taken into account only if it contains more than 20 tumor cells) (GEO: GSE125449), healthy tissues (*n* = 4) and cirrhotic tissues (*n* = 3) (GEO: GSE136103) were filtered (both gene and cell) and normalized by using the Seurat package (version 3.0) [23] in R (version 3.5.3). Genes expressed in fewer than three cells per sample were excluded, as were cells that expressed fewer than 500 genes or had a mitochondrial gene content >20% of the total UMI count. The total number of transcripts in each single-cell was normalized to 10,000. Highly variable genes were detected according to the average expression (between 0.05 and 3) and dispersion (above 0.5) of the genes, followed by data scaling (subtracting the average expression) and centering (divided by standard deviation). These variable genes were considered to account for cell-to-cell differences, and were further used for PCA. The first 20 PCs were applied for t-SNE analysis according to the eigenvalues.

### Identification of nonmalignant cell types

We extracted the transcriptome data of cells from the expression profiles of all the single cells evaluated. Similar to the total cell analysis, we first selected variable genes across cells, based on criteria of average expression (between 0.05 and 3) and dispersion (above 0.5) of the genes. Then, we performed data scaling followed by dimension reduction with PCA. The first 20 PCs were selected for t-SNE analysis. Different subclusters of cells were revealed on the t-SNE plot. We annotated the cells based on known cell lineage-specific marker genes as T cells (CD4, CD3E, CD3D, CD3G, CD8A, CD8B), B cells (CD79A, SLAMF7, BLNK, FCRL5), TECs (PECAM1, VWF, ENG, CDH5), CAFs (COL1A2, FAP, ACTA1, COL3A1, COL6A1), TAMs (CD14, CD163, CD68, CSF1R), and HPC-like (EPCAM, KRT19, PROM1, ALDH1A1, CD24).

### CNV estimation

Cells defined as endothelia, fibroblast and macrophage were used as references to identify somatic copy number variations with the R package infercnv (v0.8.2) [9]. We scored each cell for the extent of CNVs, defined as the mean of squares of CNV values across the genome. Putative tumor cells were then defined as cells with CNV signals above 0.05 and CNV correlations above 0.5.

### Constructing single-cell trajectories

We constructed a single-cell trajectory of each tumor by using the reversed graph embedding method implemented in the R Monocle package (version 2.6.3) [9]. Monocle learns the transcriptional changes in single cells and constructs a trajectory that mainly reflects the progression of cells moving from the starting state (i.e., the start of the trajectory). Monocle uses the Cell Data Set object to store single-cell gene expression data, as well as analysis results. Thus, we created a Cell Data Set object for single cells of each tumor with the parameter expression Family as negbinomial. Two major steps were then performed for single-cell trajectory construction. The first step was to detect genes that could provide important information in shaping the trajectory. To this end, we conducted PCA and t-SNE (the first 10 PCs were used) based on the genes that were expressed in at least 10% of all the cells of each tumor, and further applied the density peak clustering method to identity clusters in the t-SNE space. Differential gene expression analysis was performed among the clusters. The top 1000 derived genes were considered crucial for defining the progress of cells. The second step involved dimensionality reduction and trajectory construction with the obtained genes. The reversed graph embedding technique was applied by projecting cells to a low dimensional space while simultaneously learning smooth tree-like manifold as well as assigning cells onto the manifold.

### Diversity score

We defined a diversity score to measure the degree of transcriptional diversity and the diversity of HPCs. The diversity score was calculated based on the gene expression profiles of tumor cells and HPCs within the tumor. We employed PCA to project the original expression profiles of all malignant HPCs to the eigenvector space to derive PCs, which could capture major information and reduce noise [9]. To reduce the impact of extreme values on the diversity score calculation, we used the mean ± 3*standard deviation to detect and exclude extreme values. Among all the samples used in this study, two single cells had extreme values. Diversity scores based on the inferred CNV profiles were measured in the same way as the diversity was measured from the transcriptome as described above.

### Cellular communications

The CellPhoneDB (https://www.cellphonedb.org) package was used to search for ligand-receptor interactions. log10 of p values was calculated and plotted by Python’s pyplot.scatter (https://matplotlib.org/3.2.1/api/_as_gen/matplotlib.pyplot.scatter.html). A more in-depth analysis of the interaction between cells was conducted using NicheNet (https://github.com/saeyslab/nichenetr) based on public databases such as KEGG, ENCODE and PhoshoSite. Using our cell type labeled whole atlas Seurat object, receiver cell type is specified as malignant cells sender cell type as TAFs whereas condition of interest is ‘Tumor’ as compared to ‘Normal’ region in *nichenet_seuratobj_aggregate*. Nichenet heatmap are generated using *nichenet_output$ligand_activity_target_heatmap* [24]. Nichenet tutorials are found in the GitHub page.

### Statistical analysis

Statistical analysis was performed using SPSS software version 20.0 (SPSS, Chicago, IL) and Python 3.6. Data are presented as the means ± S.E.M. Differences were analyzed by Student’s t-test and one-way ANOVA. Tumor incidence (%) was analyzed by Fisher’s exact test. The Kaplan-Meier method was used to calculate the survival rate and the Log-rank test was used to determine the significance of differences. Correlations between the expression levels of genes were calculated using Pearson correlation, and a linear model was built to fit the data and test the significance and was plotted as a trend-line with confidence intervals. A *P*-value of < 0.05 was considered statistically significant.

## Results

### Landscape of transcriptional diversity of liver cancer patients

**Fig. 1:**
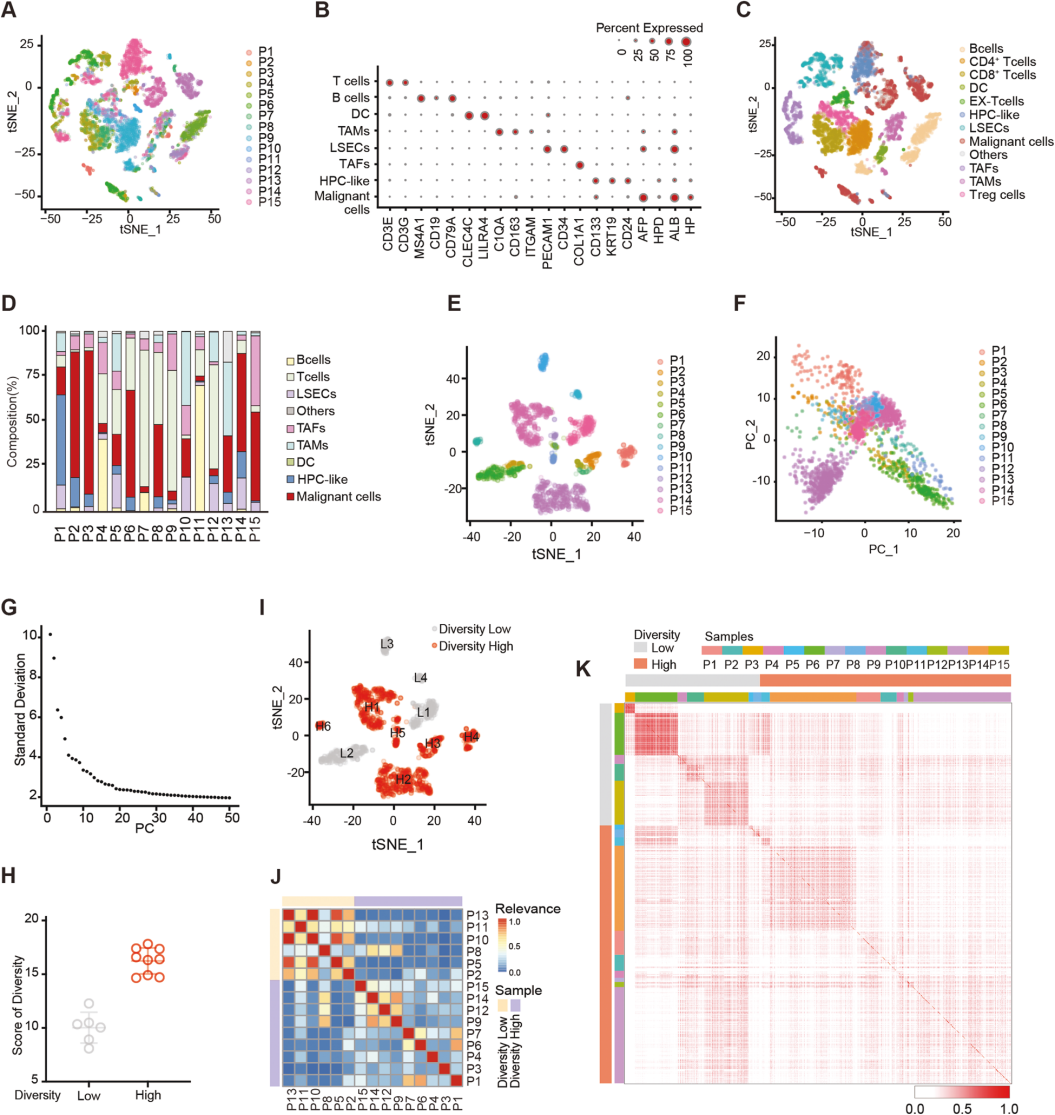
Transcriptomic diversity of liver cancer patients. **A** t-SNE plot of all single cells from 15 primary liver cancer patients (indicated by colors). **B** Bubble chart shows the expression of canonical marker genes for annotating the cell types. **C** t-SNE plot of cell types from tumors (indicated by colors). Cells were annotated as T cells, B cells, TAFs, TAMs, LSECs, and HPC-like based on known lineage-specific marker genes (from **B**). The malignant cells were identified by infering CNV. **D** Stacked bar plots showing the cell composition of the 15 samples. **E** t-SNE plot of tumor cells from 15 tumors (indicated by colors). **F** Principal-component analysis (PCA) of tumor cells from 15 tumors. **G** PCA of tumor cells. Eigenvalue corresponding to each PC. **H** Transcriptomic diversity score of tumor samples according to the median value of diversity. Data are presented as the means ± SEM. **I** tumor cells t-SNE plot of tumor cells from the diversity-low (gray dots, L1-4) and diversity-high (red dots, H1-6) groups. **J**, **K** Pairwise correlation of all tumor cells from 15 liver cancer patients. Each pixel in the heatmap represents a correlation of two cells (the corresponding row and column). At least two independent experiments were performed for all data. For curve Figures and bar Figures, data are presented as means ± SD. Unpaired Student’s t-tests were used for comparing two variables. One-way ANOVA was used for multiple variables comparison.

To examine the transcriptional diversity of liver cancer, we observed single-cell RNA sequencing (scRNA-seq) by 10X genomics profiles of 15 patients with liver cancer [9] (GSE125449) (Fig. 1A). Single-cell transcriptomes for 8460 cells were analyzed after quality control. We applied principal-component analysis (PCA) on the genes with the greatest variability (*k* = 2 244) across all cells. With the linearly uncorrelated principal components (PCs) (*k* = 23), we performed t-distributed stochastic neighbor embedding (t-SNE) analysis. We discriminated 1797 tumor cells by inferring large-scale chromosomal copy-number variations (CNVs) [7]. Then, nontumor cells were annotated based on the known cell lineage-specific marker genes unique to T cells, B cells, tumor-associated fibroblasts (TAFs), tumor-associated macrophages (TAMs), liver sinusoidal endothelial cells (LSECs), and cells expressed hepatic progenitor cells (HPC-like) markers (Fig. 1B, C). The cell composition was highly heterogeneous among these cases (Fig. 1D).

To further determine the transcriptomic diversity of tumor cells, we extracted all the tumor cells and calculated tumor cell-specific transcriptomic diversity scores (Fig. 1E). We defined the diversity score to measure the degree of heterogeneity of tumor cells [9]. PCA was used to project the original expression profiles of all tumor cells into the feature vector space to obtain PC, which can capture main information and reduce noise (Fig. 1F, G). We calculated the mass point of each sample (the arithmetic mean of all tumor cells PC in the corresponding tumor). We defined the diversity score of all tumor cells as the average distance of them to the centroid (Fig. 1E-G), and divided them into diversity-high and diversity-low groups (Fig. 1H, I). Heterogeneity in tumor cells within and between tumors could be further revealed by correlation analysis of all tumor cells. We found that there was high heterogeneity among cases (Fig. 1J), while similarities of the same type of tumor vary from tumor to tumor (Fig. 1K). For instance, the diversity-low group had a relatively homogeneous tumor cell population, while the diversity-high group the opposite, which suggested that the transcriptomic diversity of the high group was more significant (Fig. 1K). Altogether, our data demonstrated that the heterogeneity of tumor cells in liver cancer varied substantially between and within tumors.

### Tumor cells of transcriptomic diversity-high group with a greater capacity of stemness

**Fig. 2:**
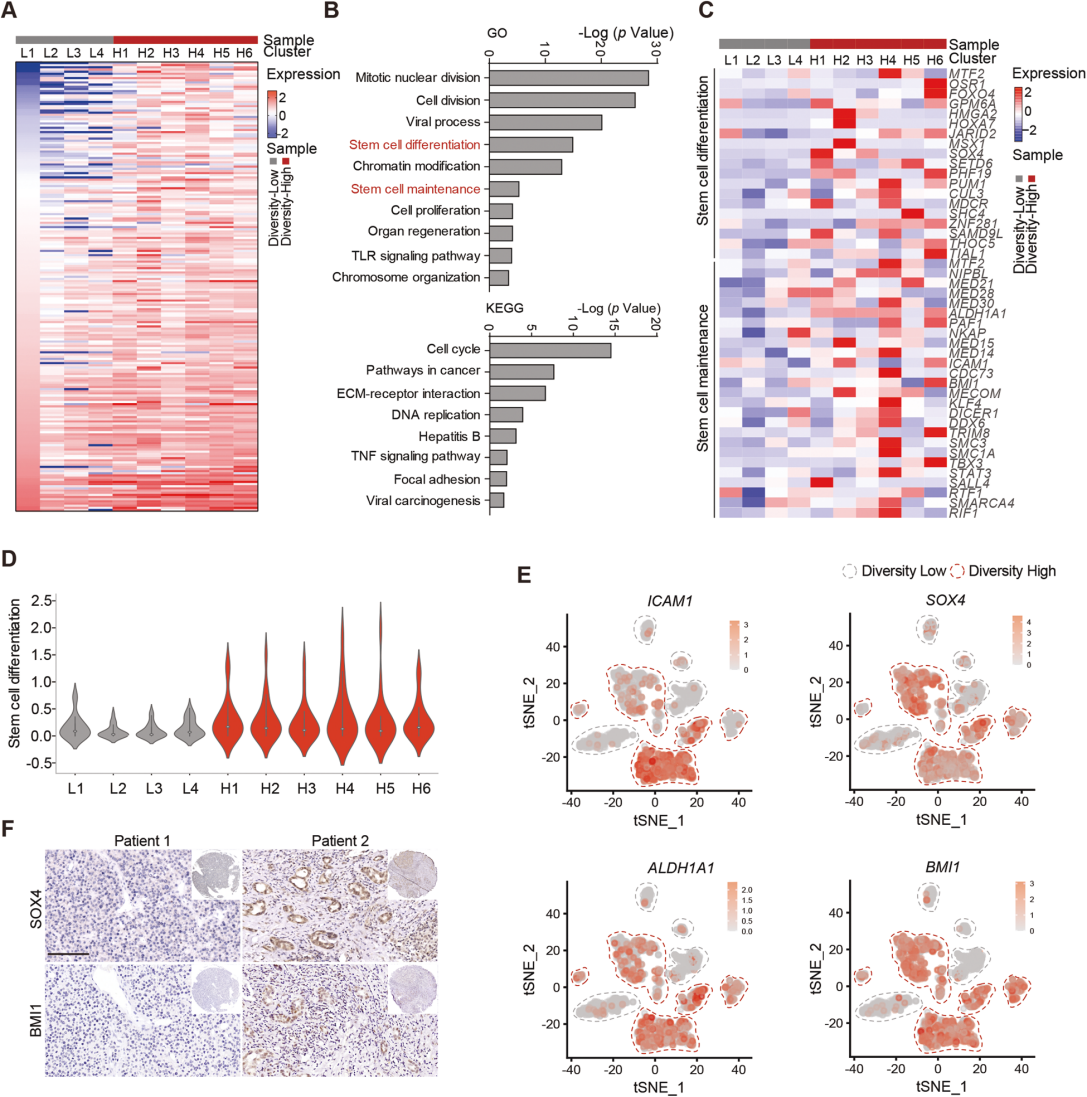
Tumor cells in diversity-high group possess stemness characteristics. **A** Heatmap of upregulated genes of tumor cells in the diversity-high groups compared to diversity-low groups. **B** Gene Ontology (GO) and Kyoto Encyclopedia of Genes and Genomes (KEGG) analysis of upregulated genes of diversity-high groups in Fig. 2A. **C** Heatmap of genes in diversity-high and diversity-low groups belong to stem cell differentiation and stem cell maintenance gene sets. **D** Violin plot of stem cell differentiation scores for the clusters in Fig. 2A. **E** t-SNE plots showing the expression levels of *ICAM1, SOX4, ALDH1A1* and *BMI1* from the diversity-high and diversity-low groups of tumor cells. **F** Staining of SOX4 and BMI1 in tumor tissue from liver cancer patients. Bar, 100μm. At least two independent experiments were performed for all data. For curve Figures and bar Figures, data are presented as means ± SD. Unpaired Student’s t-tests were used for comparing two variables. One-way ANOVA was used for multiple variables comparison.

To gain insight into the molecular characteristics of transcriptomic diversity, we analyzed the upregulated genes in the diversity-high group (H1-H6) compared with the diversity-low group (L1-L4) (Figs. 1I and 2A). Gene Ontology (GO) and Kyoto Encyclopedia of Genes and Genomes (KEGG) pathway enrichment analysis revealed that multiple biological processes, including stem cell differentiation, stem cell maintenance and DNA replication, were more active in the diversity-high group (Fig. 2B). Additionally, stemness-related genes were upregulated in the diversity-high group (Fig. 2C). Besides, gene set variation analysis (GSVA) suggested that the diversity-high group had high scores for stem cell differentiation (Fig. 2D). Accordingly, the expression profiles of stemness-related markers, such as *ICAM1* [25], *SOX4* [26], *ALDH1A1* [27] and *BMI1* [28], were higher in the diversity-high group (Fig. 2E). Moreover, we further performed validation at the histological level. Compared with patient 1, elevated expression of SOX4 and BMI1 were observed in patient 2 (higher heterotypic of tissue structure and cell morphology, indicating a high diversity) by Immunohistochemistry (IHC) analysis (Fig. 2F). Taken together, these results suggested that tumor cells with high transcriptomic diversity exhibited a greater capacity of stemness.

### TAF is predicted to drive the transcriptional diversity of tumor cells via ECM-receptor interaction

**Fig. 3:**
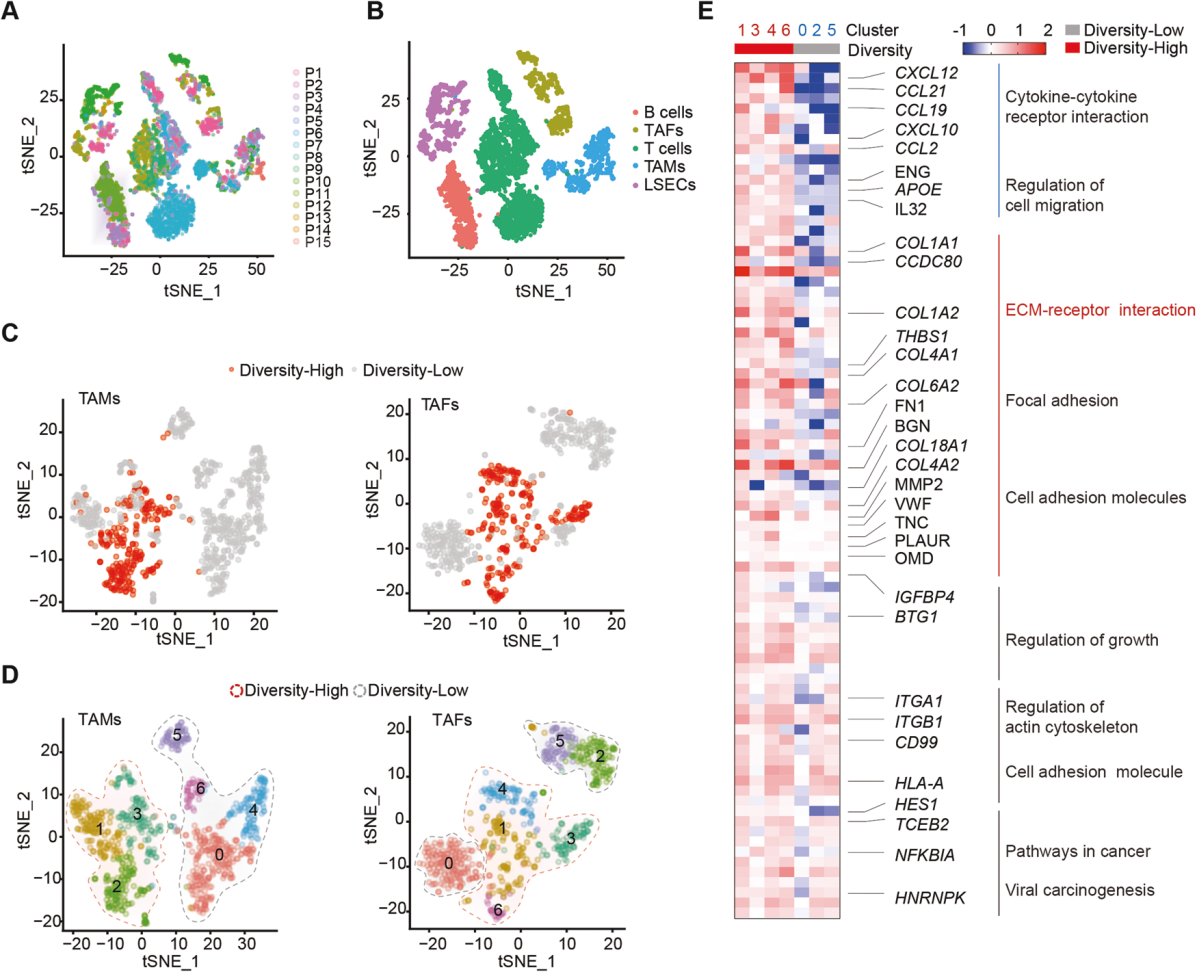
Composition of tumor microenvironment derived from diversity-low and diversity-high groups. **A**, **B** t-SNE plot of nontumor cells from 15 tumors (indicated by colors). **C** t-SNE plot of TAFs, TAMs and LSECs from the diversity-low (gray dots) and diversity-high (red dots) groups. **D** t-SNE plot showing clusters identified by integrated analysis of TAMs and TAFs in diversity-low and diversity-high tumors. **E** Heatmap and KEGG analysis of genes in clusters of TAFs. At least two independent experiments were performed for all data. For curve Figures and bar Figures, data are presented as means ± SD. Unpaired Student’s t-tests were used for comparing two variables. One-way ANOVA was used for multiple variables comparison. See also Fig. S1.

To explore the crucial microenvironmental factors that induce transcriptomic diversity, we extracted nonparenchymal cells from all samples (Fig. 3A, B). Nonparenchymal cells derived from diversity-high and diversity-low tumors differ in their transcriptomic profiles (Fig. 3C, D and Figs. S1A-D). The difference was further evident in TAFs and TAMs between diversity-high and diversity-low groups (Fig. 3C). In contrast, a nonsignificant difference in T cells was shown between the two groups (Fig. S1A-C). We further analyzed differentially expressed genes between the diversity-high and diversity-low tumor groups and observed significant changes in the expression patterns of TAFs, B cells and LSECs (Fig S1E). Since TAFs and TAMs showed a pivotal role in the development of liver cancer, we did further analysis for clusters in them (Fig. 3D). A significant distinction of TAFs was observed (Fig. 3E), yet not in TAMs (Fig S1F). KEGG enrichment analysis revealed that the differential gene enriched in the ECM-receptor interaction pathway (Fig. 3E). Taken together, these results indicated that ECM-receptor interaction might be involved in the development of transcriptomic diversity of tumor cells in liver cancer.

### The linkage between TAF and tumor cells is related to ECM-receptor interaction

**Fig. 4:**
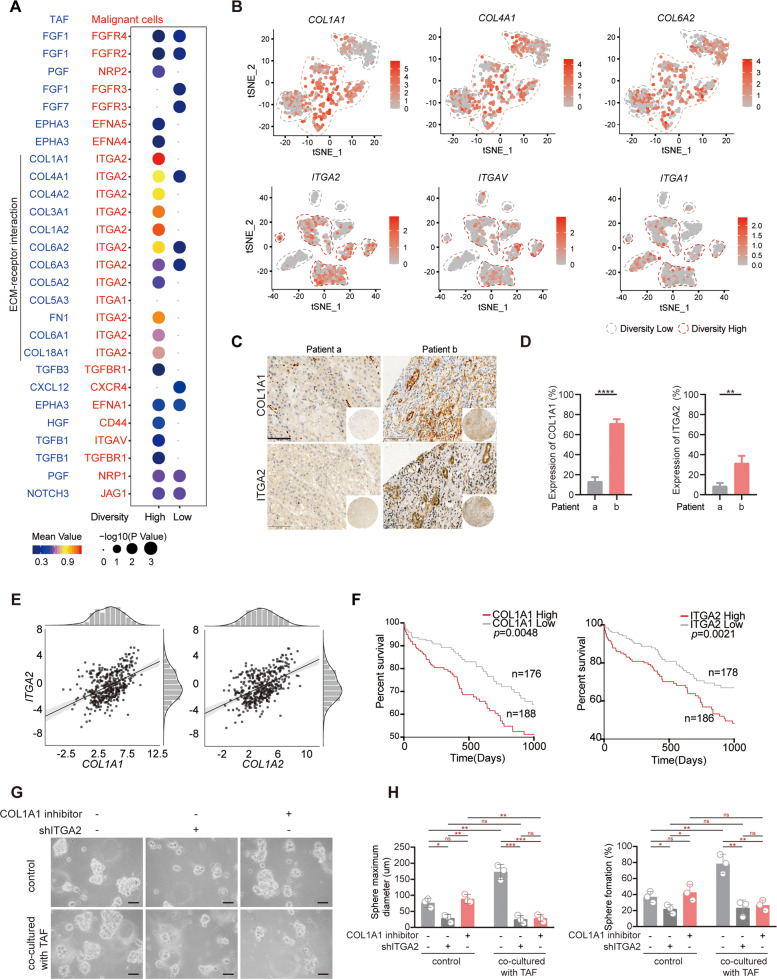
Crosstalk between TAF and Tumor cells. **A** The bubble plot of ligands and receptors involved in significant L-R pairs between TAFs and tumor cells from diversity-high tumors. **B** t-SNE plots showing the expression profiles of COL1A1, COL4A1, and COL6A2 of TAF in the diversity-high and diversity-low groups. The level of ITGA2, ITGAV, and ITGA1 from the diversity-high and diversity-low groups of tumor cells. **C** Representative cores of COL1A1 and ITGA2 staining in the tissue microarray. Bar, 100 μm. **D** Positivity for the expression of COL1A1 and ITGA2 in the tissue microarray. **E** The correlation between COL1A1/2 and ITGA2 in TCGA patients. **F** Kaplan-Meier survival analysis of COL1A1 and ITGA2 at high or low levels in tumors from the TCGA database. **G**, **H** The capacity of colony formation was detected by sphere formation assays in Huh7 cell lines. Cells with or without co-cultured with CAFs were knocked down ITGA2 or treated with COL1A1 inhibitor for 3 days. Bar, 100 µm. At least two independent experiments were performed for all data. For curve Figures and bar Figures, data are presented as means ± SD. Unpaired Student’s t-tests were used for comparing two variables. One-way ANOVA was used for multiple variables comparison. *P < 0.05; **P < 0.01; ***P < 0.001; n.s, no significance in comparison with control group. See also Fig. S2.

Given the above observations that TAF might contribute to the high heterogeneity of tumor cells, we next focused on the interactions between TAF and tumor cells. A set of ECM-related ligand-receptor (L-R) pairs exhibited a higher gene detection sensitivity in diversity-high subsets, such as *COL1A1/2-ITGA2/V, COL4A1/2-ITGA2, COL6A1/2-ITGA2*, and *FN1-ITGA2/V* (Fig. 4A). We then analyzed the expression profiles of *COL1A1*, *COL4A1*, and *COL6A2* in TAF and *ITGA2*, *ITGAV*, and *ITGA1* in tumor cells. In Fig. 4A, the level of *COL1A1* and *ITGA2* were highest in the candidates, suggesting *COL1A1-ITGA2* possesses the potential for mediating the crosstalk of TAF and tumor cells in high transcriptomic diversity groups (marked by red dotted lines) (Fig. 4B). Similarly, IHC staining showed the physical juxtaposition of COL1A1 and ITGA2 in tumor tissue from patient b, which exhibited marked atypia (Fig. 4C, D). Based on the analysis of 367 liver cancer patients from The Cancer Proteome Atlas (TCGA) database, the COL1A1 and COL1A2 signatures showed modest correlation with ITGA2 (*R* = 0.49, *p* < 2.2e-16, *R* = 0.51, *p* < 1.7e-12, Pearson’s correlation) (Fig. 4E). Kaplan-Meier analysis showed a clear association of high levels of COL1A1 and ITGA2 with poor prognosis of liver cancer patients (all *P* < 0.05) (Fig. 4F). To better demonstrate the role of COL1A1-ITGA2 interaction on the transcriptomic diversity of tumor cells, we now performed co-culture experiments of ITGA2 knockdown tumor cells and COL1A1 silenced TAFs. Since the established TAF line is not available, we first harvested primary TAFs from fresh tumor tissue, and microscopic evaluation of cell cultures exhibited the elongated and fibroblast-like morphology of TAFs (Fig S2A). Then, sphere formation demonstrated that the capacity of spheres formation of tumor cells obviously increased in co-cultured with TAFs group, and were markedly reduced after knocking down ITGA2. Additionally, the COL1A1 inhibitor only decreased spheres formation of tumor cells when they co-cultured with CAFs (Fig. S2B and Fig. 4G, H). Altogether, our data demonstrated that TAFs might regulate the diversity of tumor cells via various cellular interactions, especially COL1A1-ITGA2.

### YAP-Signaling is involved in transcriptional diversity of tumor cells

**Fig. 5:**
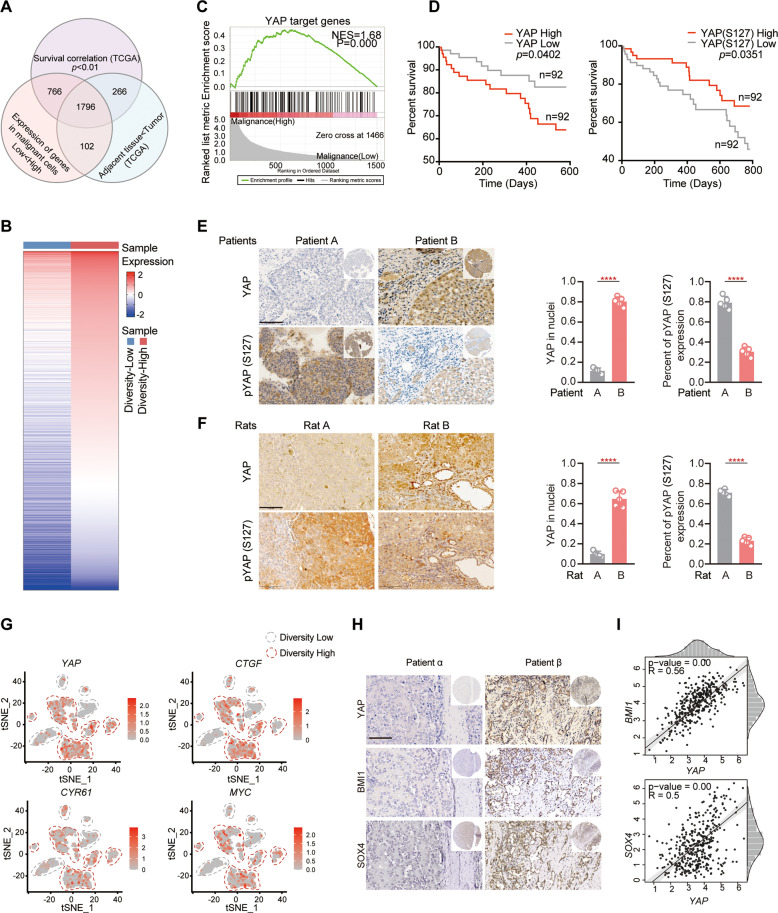
YAP signaling with the potential to involve in the diversity of liver cancer. **A** Integrative analysis of data from the scRNA-seq screen with publicly available databases of liver cancer from the TCGA. **B** Heatmap of genes with increased expression in tumor cells from diversity-high group. The color of each group represents the average gene abundance. **C** Gene set enrichment analysis (GSEA) analysis showing significant positive enrichment of YAP signaling with upregulated genes in tumor cells from the diversity-high group. **D** Kaplan-Meier survival analysis of patients with YAP at high or low protein levels from The Cancer Proteome Atlas (TCPA). **E**, **F** Staining of YAP in tumor tissues from liver cancer patients and rats treated with DEN up to 16 weeks. Bars, 100 µm. **G** t-SNE plots showing the expression levels of *YAP*, *CTGF*, *CYR61* and *MYC* in tumor cells of diversity-high and diversity-low groups. **H** Representative cores of YAP, BMI1, and SOX4 staining in the tissue microarray. Bar, 100 μm. **I** Pearson correlation analysis of the mRNA levels between BMI1/SOX4 and YAP in liver cancer patients from the TCGA database. At least two independent experiments were performed for all data. Quantified data are presented as the means ± SD. Unpaired Student’s *t* tests were used for comparing two variables and one-way ANOVA was used for comparing multiple variables. *P < 0.05; **P < 0.01; ***P < 0.001; n.s, no significance in comparison with the control group. See also Fig. S3.

To dissect the molecular mechanism of transcriptomic diversity, we analyzed the significantly different genes in the high and low diversity groups. Combining the genes increased in tumor tissue and associated with poor prognosis in liver cancer data from the TCGA database, we identified 1796 candidates upregulated with high confidence in the diversity-high group (Fig. 5A, B). Gene set enrichment analysis (GSEA) revealed that a group of YAP-targeted genes was upregulated among these genes (Fig. 5C). These observations indicated that YAP-signaling probably participated in the transcriptional diversity of tumor cells.

To determine the role of activated YAP in transcriptional diversity, we observed the protein levels of YAP and phosphorylated YAP (S127) in 367 patients in The Cancer Proteome Atlas (TCPA). Our results showed a clear association of high protein levels of YAP with poor prognosis of liver cancer patients, whereas, low protein levels of phosphorylated YAP (S127) were associated with poor prognosis (Fig. 5D). We further evaluated YAP and phosphorylated YAP (S127) protein levels in liver cancer tissues from both patients and animals. IHC analysis showed that the nuclear accumulation of YAP in tumor cells was significantly increased in the patient’s tumor tissues which had obvious heterotypic structure (Fig. 5E). A similar result was shown in the rats treated with DEN for 16 weeks (Fig. 5F). Conversely, phosphorylated YAP (S127) occurred less frequently in the tumor tissues with obvious heterotypic both of patients and rats (Fig. 5E, F). Next, we analyzed the expression profiles of *YAP* and target genes at the single-cell level. Except for *CYR61*, the expression of *YAP*, *CTGF* and *MYC* were significantly elevated in tumor cells from the diversity-high group (Fig. 5G). Besides, given the tumors with high transcriptional diversity had a stronger potential for stemness as mentioned in Fig. 2, and YAP-signaling is well known to regulate stem cell homeostasis [12]. Reasonably, we assumed that YAP activation might participate in the formation of transcriptional diversity by promoting stemness. To further elucidate this assumption, we analyzed the protein expression of YAP, BMI1, and SOX4 (stemness-related genes, depicted in Fig. 2E) in tumor tissue. IHC assays revealed increased co-localization between YAP and BMI1 in patient β. Histologically, patient β architecture was disorganized with heterotypic tumor cells. A similar result was shown in YAP and SOX4 (Fig. 5H). We further detected the staining of YAP, BMI1, and SOX4 in the TCGA database. There was a positive correlation between BMI1/SOX4 and YAP in liver cancer patients (*R* = 0.56, *P* < 0.05, *R* = 0.5, *P* < 0.05, Fig. 5I). Although ALDH1A1 was highly expressed in the diversity-high group (depicted in Fig. 2E), no correlation between ALDH1A1 and YAP in liver cancer specimens was observed (Fig. S3). Together, these observations suggested that YAP-signaling might mediate the transcriptional diversity of tumor cells in liver cancer.

### Collagen/ITGA2-stimulated YAP activation is associated with transcriptional diversity via promoting the stemness

**Fig. 6:**
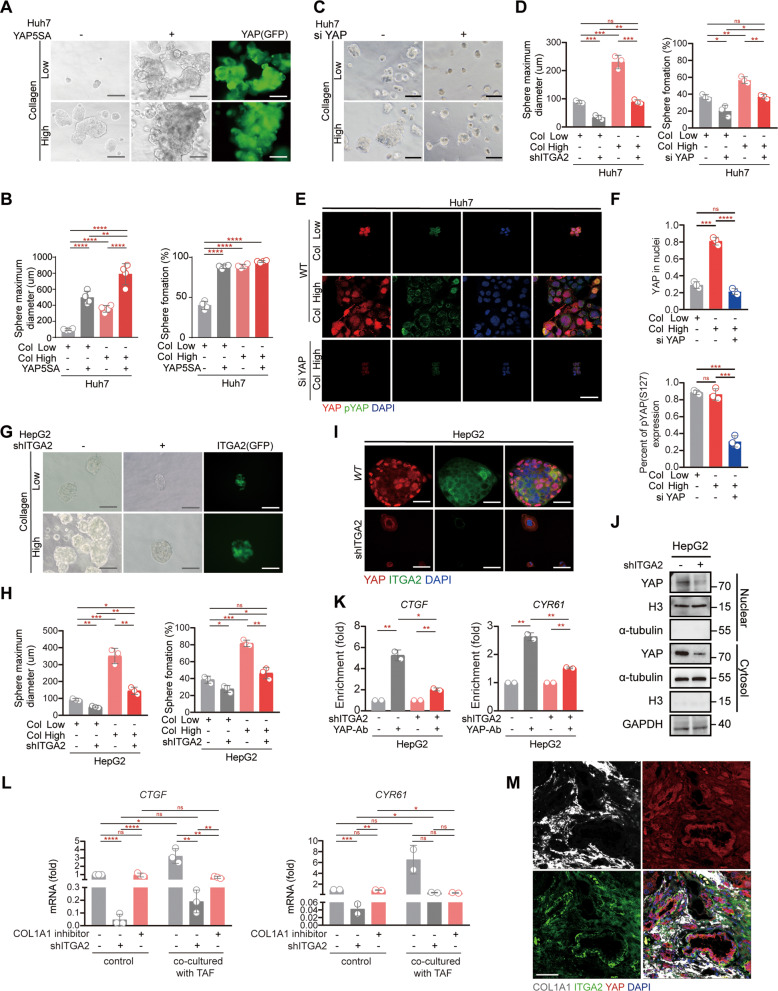
Collagen-mediated YAP activation is predicted to relate with diversity by enhancing stemness of tumor cells. **A**, **B** The capacity of colony formation was detected by sphere formation assays in Huh7 (liver cancer cell line). Cells with or without YAP overexpression (GFP-labeled) were treated with low (1 µL collagen with 3 µL DMEM) or high (collagen without DMEM) concentrations of collagen for 3 days. Bar, 200 µm. **C**, **D** The capacity of colony formation was detected by sphere formation assays in Huh7 cells. Cells with or without YAP knockdown were treated with low (1 µL collagen with 3 µL DMEM) or high (collagen without DMEM) concentrations of collagen for 3 days. Bar, 200 µm. **E**, **F** Staining of YAP in Huh7 cells cultivated with low or high concentrations of collagen for 3 days, 200 µm. **G**, **H** The capacity of colony formation was detected by sphere formation assays in HepG2 (liver cancer cell lines). Cells with or without ITGA2 knockdown (GFP-labeled) were treated with low (1 µL collagen with 3 µL DMEM) or high (collagen without DMEM) concentrations of collagen for 3 days. Bar, 200 µm. **I** Staining of YAP and ITGA2 in HepG2 cells (with or without ITGA2 knockdown) for 3 days, 200 µm. **J** Subcellular localization of YAP in WT and ITGA2-knockdown HepG2 cells. **K** ChIP experiment of CTGF and CYR61 performed with YAP antibody in HepG2 cells knockdown ITGA2. **L** mRNA levels of CTGF and CYR61 in WT and ITGA2-knockdown Huh7 cells with or without co-cultured with TAFs. **M** Staining of COL1A1, ITGA2, and YAP in tumors from liver cancer patients. Bars, 100 µm. Quantified data are presented as the means ± SD. Unpaired Student’s *t* tests were used for comparing two variables and one-way ANOVA was used for comparing multiple variables. At least two independent experiments were performed for all data. *P < 0.05; **P < 0.01; ***P < 0.001; n.s, no significance in comparison with the control group. See also Fig. S4.

Based on the above observation of COL1A1 probably mediated the transcriptional diversity via the interaction between TAFs and tumor cells (Fig. 4A), we cultured tumor cells in medium with different dilutions of collagen and detected their stemness by sphere formation. High concentrations of collagen obviously increased the capacity of spheres formed (Figs. 6A, B, S4A, B). The low levels of collagen abrogated sphere formation, whereas overexpression of YAP fully rescued the ability of tumor cells to form spheres (Fig. 6A, B, S4A and S4B). Conversely, the knockdown of YAP inhibited the ability of tumor cells to form spheres (Fig. 6C, D). Further immunofluorescence analysis showed that the YAP nuclear localization was increased in the high collagen group, whereas not show altered phosphorylated YAP (S127) levels in the high collagen stimulated (Figs. 6E, F, S4C, D). Additionally, for YAP, both nuclear and cytoplasmic immunopositivity were denervated in the YAP-knockdown group, yet, these cells were still able to phosphorylate YAP (S127) (Figs. 6E, F, S4C, D). Collectively, these results indicated that collagen-induced YAP activation promotes stemness potentially contributed to the high diversity of tumor cells.

Combine our above results with previous studies that mechanical stress promoted the activation of YAP through the integrin protein receptor family (ITGA2/V) [29–31], we hypothesized that TAF-secreted COL1A1 interacts with ITGA2 potentially activated YAP in tumor cells, which possibly contributed to the transcriptional diversity thought enhancing stemness of tumor cells in liver cancer. To test this hypothesis, we observed the sphere formation ability of tumor cells in different concentrations of collagen after knocking down ITGA2. The deficiency of ITGA2 obviously inhibited both size and efficiency of tumorspheres (Fig. 6G, H). Moreover, immunofluorescence analysis and nuclear-cytoplasmic fractionation assay showed that the nuclear localization of YAP was decreased in the ITGA2 knockdown cells (Fig. 6I, J). Additionally, the YAP-binding affinity to the promoters of *CTGF* and *CYR61* was greatly reduced under the ITGA2 deficiency condition (Fig. 6K). Then, we probed the interaction between COL1A1, ITGA2, and YAP. In co-culture system of ITGA2 knock-down tumor cells and COL1A1 silenced TAFs, the transcription of CTGF and CYR61 was dramatically reduced in ITGA2-depleted cells, whereas, it was elevated in co-cultured with TAFs and markedly decreased after being treated with the COL1A1 inhibitor (Fig. 6L). The IHC experiments showed that the tumor cells highly accumulated YAP in the nucleus were surrounded by TAF labeled by α-SMA (Fig S4E). Next, to verify the correlation between COL1A1-ITGA2 and YAP activation, we detected the expression profiles of *COL1A1*, *ITGA2*, *YAP*, and *CTGF* from the TCGA database. There was a positive correlation between COL1A1/ITGA2 and YAP/CTGF in liver cancer patient (*R2* = 0.35, *P* < 1.1e-12, *R*2 = 0.49, *P* < 2.2e-15, Fig S4F). Additionally, physical juxtaposition staining patterns were observed for COL1A1, ITGA2, and YAP in diversity-high tumor tissues (Fig. 6M), indicating an interaction between TAF and tumor cells mediated by COL1A1-ITGA2 and YAP-signals in diversity-high liver cancer patients. In summary, COL1A1-ITGA2 was predicted to mediate YAP activation, which was potentially involved in transcriptional diversity via promoting the stemness of tumor cells in liver cancer.

### YAP-signaling possibly mediated transcriptional diversity by elevating the stem target genes

**Fig. 7:**
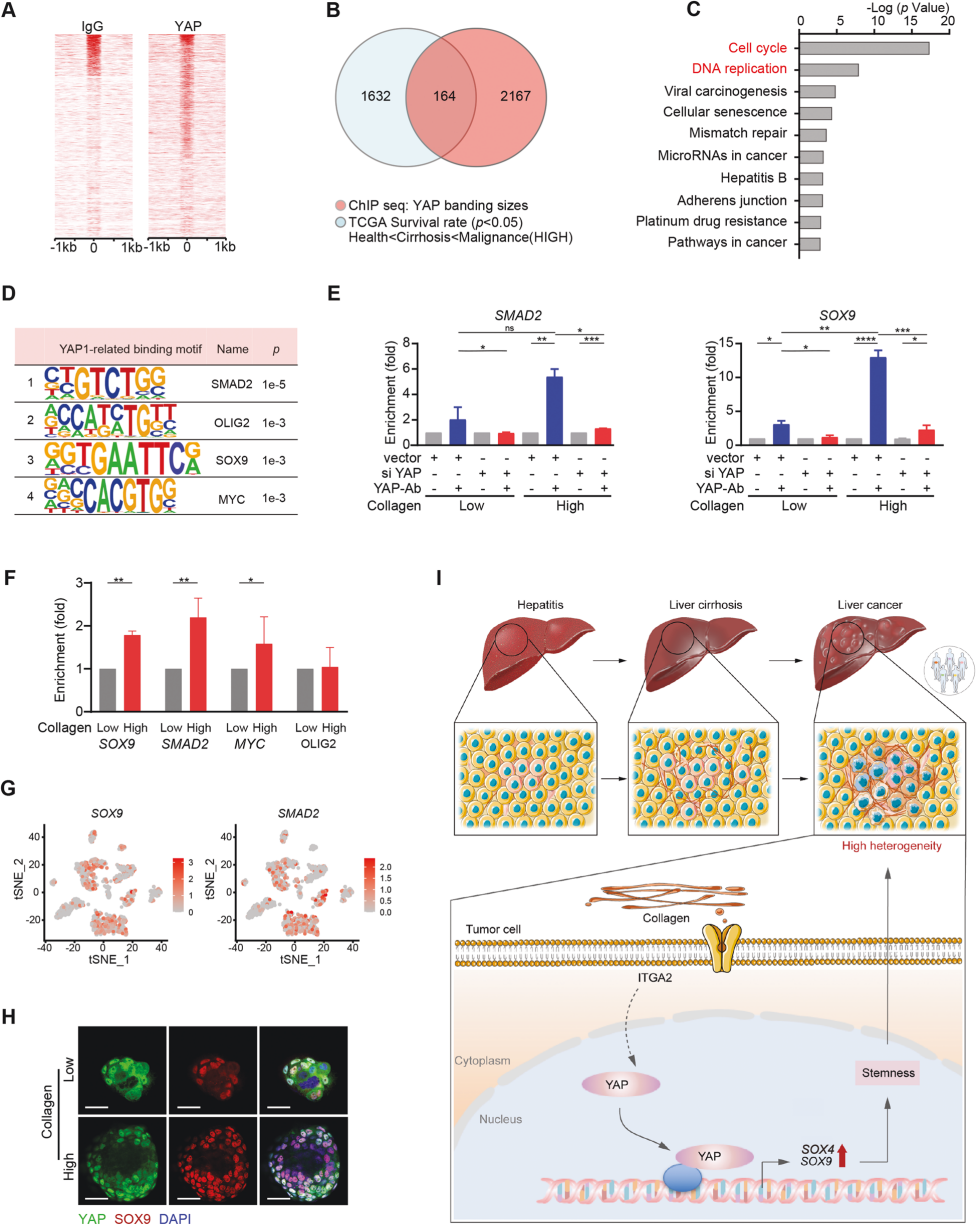
Collagen-mediated the target genes of YAP facilitated the stemness of tumor cells. **A** YAP and IgG peaks are ranked from the strongest to weakest signal. **B** Integrative analysis combining the data from ChIP-seq with the 1796 genes screened in Fig. 4A. **C** KEGG analysis performed with the 164 genes identified in (**B**). **D** Motif analysis of YAP ChIP-seq. **E** ChIP experiment performed with YAP antibody in Huh7 cells treated with different concentrations of collagen for 3 days. **F** mRNA levels of *MYC, SOX9, SMAD2,* and *OLIG2* in Huh7 cells treated with different concentrations of collagen for 3 days. **G** t-SNE plots showing the expression levels of *SOX9 and SMAD2* in tumor cells. **H** Nuclear colocalizations of YAP and SOX9 in sphere formation assays of Huh7 cells. Bars, 100 µm. **I** Schematic outline showing that TAF Involves in Transcriptional Diversity via Activating YAP Signaling in Liver cancer. At least two independent experiments were performed for all data. Quantified data are presented as the means ±SD. Unpaired Student’s *t* tests were used for comparing two variables and one-way ANOVA was used for comparing multiple variables. At least two independent experiments were performed for all data. *P < 0.05; **P < 0.01; ***P < 0.001; n.s, no significance in comparison with the control group.

To further explore the molecular mechanism of YAP target gene-mediated transcriptional diversity, we performed chromatin immunoprecipitation followed by sequencing (ChIP-seq). Analysis of the distribution of YAP-binding sites relative to genes annotated in the human genome revealed that most of the peaks were located close (<1 kb) to the transcription start sites (TSS, Fig. 7A). Then, we intersected the 1796 genes identified (blue, Fig. 5A) and YAP-binding sites (2331, red), and identified 164 confidence candidates (Fig. 7B). KEGG pathway enrichment analysis revealed that these candidates were involved in multiple biological processes, including cell cycle and DNA replication (Fig. 7C). Moreover, YAP was enriched at the promoter regions of several key factors involved in the stemness of tumor cells, such as *SOX9* [32]*, SMAD2/4* [33], *OLIG2* [23], and *MYC* [34] (Fig. 7D). These results indicated that YAP target genes might be involved in transcriptional diversity through promoting stemness.

ChIP-qPCR assays confirmed that increased YAP binding at *SMAD2 and SOX9* promoter regions compared to that of control IgG, and the binding was greatly enhanced upon cell culture in the presence of a high concentration of collagen (Fig. 7E). Notably, this enhancement was suppressed after YAP knockdown (Fig. 7E). Moreover, qPCR assays manifested that higher collagen levels significantly promoted the transcription of *MYC* [35]*, SOX9* [36], and *SMAD2* (Fig. 7F). Consistently, *SOX9* and *SMAD2* were significantly elevated in tumor cells from the diversity-high group (Fig. 7G). Immunofluorescence assays revealed strong co-localization between YAP and SOX9, which was enhanced in cells treated with high concentrations of collagen (Fig. 7H). Collectively, these results indicated that *SOX9* and *SMAD2* acted downstream of YAP-signaling potentially increase transcriptional diversity in liver cancer patients.

## Discussion

Liver cancer, a strikingly heterogeneous disease, has brought outstanding challenges to the treatment of patients. Transcriptional diversity may be practical to overcome this difficulty. Heterogeneity is mainly based on transcriptome sequencing of tissues, and the accompanying analysis noise limits its accuracy. Single-cell sequencing (scRNA-seq) can maximize unbiased information to explore the transcriptional diversity at the single-cell level. Although the use of scRNA-seq to identify tumor heterogeneity in liver cancer is rapidly increasing [9], the characterization of parenchymal cells remains unknown. Here, we measured the diversity score to analyze the degree of transcriptional diversity by using principal components (PCs), and analyzed the relationship between tumor and nontumor cells. Our findings reveal that the tumor microenvironment (TME) increases transcriptional diversity in liver cancer, in which tumor-associated fibroblast (TAF) contributes to the transcriptional diversity and may impact tumor progression.

The source of tumor heterogeneity is the poor TME. Studies have demonstrated that stromal cells are as highly heterogeneous as cancer cells [37]. We discovered that the distribution and molecular characteristics of TAF are highly consistent with the transcriptional diversity of tumor cells. TAF modifies the composition of the TME and the development of cancer [38], and both TAF secreted cytokines (such as CCl2, CXCL1 [19], and IL-6 [39]) and ECM [40] could drive the stemness of tumor cells. Additionally, ECM is crucial in the formation of tumor heterogeneity [41]. However, the underlying molecular mechanism of ECM-mediated transcriptional diversity remains unclear. In this study, TAF triggers the accumulation of collagen, while the corresponding receptors occurred in diversity-high tumor cells subsets. These results suggest that the evolution of the TME is involved in transcriptional diversity in liver cancer patients, which is significant to further explore the formation mechanism of heterogeneity in liver cancer. Besides, fibroblasts are essential for the development of liver cirrhosis and its transformation to liver cancer [10]. Therefore, our study suggests that intervention in liver cirrhosis might be beneficial to reduce liver cancer heterogeneity.

The transcriptional regulator YAP is remarkedly required for cell proliferation, tissue regeneration, and tumorigenesis [42]. However, it’s unclear whether the activation of YAP is related to the heterogeneity. Further analysis of the single-cell dataset revealed that the activation of YAP-signaling was associated with transcriptional diversity in liver cancer. Moreover, we observed that collagen-triggered YAP activation feasibly facilitated the transcriptional diversity via enhancing the stemness of tumor cells. Here, we verified for the first time at the single-cell level the essential role of activating YAP-signaling involves in the transcriptional diversity of liver cancer, offering potential molecular mechanisms for individualized treatment.

Summarily, we show that TAF drives transcriptional diversity of liver cancer via collagen-stimulated YAP-signaling hyperactivation. We find for the first time that the stemness of tumor cells is related to the transcriptional diversity of liver cancer at the single-cell level. We use single-cell data and observe that transcriptional diversity of tumor cells arises from the evolutionary selection of the dynamic TME in liver cancer patients. In the process, COL1A1-ITGA2-mediated YAP-signaling activation possibly regulates transcriptional diversity via enhancing the stemness of tumor cells (Fig. 7I). Our research provides a theoretical basis and molecular mechanism for exploring individualized liver cancer treatment.

## Supplementary information

Original Data File

Related 2. EXPERIMENTAL PROCEDURES

Supplemental figures

## Data Availability

All data needed to evaluate the conclusions in the paper are present in the paper. Additional data related to this paper may be requested from the corresponding author.

## References

[1] Maley CC, Aktipis A, Graham TA, Sottoriva A, Boddy AM, Janiszewska M (2017). Classifying the evolutionary and ecological features of neoplasms. Nat Rev Cancer.

[2] McGranahan N, Swanton C (2017). Clonal Heterogeneity and Tumor Evolution: Past, Present, and the Future. Cell.

[3] Medema JP (2013). Cancer stem cells: The challenges ahead. Nat Cell Biol.

[4] Goodell MA, Nguyen H, Shroyer N (2015). Somatic stem cell heterogeneity: diversity in the blood, skin and intestinal stem cell compartments. Nat Rev Mol Cell Bio.

[5] Jusakul A, Cutcutache I, Yong CH, Lim JQ, Huang MN, Padmanabhan N (2017). Whole-Genome and Epigenomic Landscapes of Etiologically Distinct Subtypes of Cholangiocarcinoma. Cancer Disco.

[6] Boyault S, Rickman DS, de Reynies A, Balabaud C, Rebouissou S, Jeannot E (2007). Transcriptome classification of HCC is related to gene alterations and to new therapeutic targets. Hepatology..

[7] Chaisaingmongkol J, Budhu A, Dang H, Rabibhadana S, Pupacdi B, Kwon SM (2017). Common Molecular Subtypes Among Asian Hepatocellular Carcinoma and Cholangiocarcinoma. Cancer Cell.

[8] Seehawer M, Heinzmann F, D’Artista L, Harbig J, Roux PF, Hoenicke L (2018). Necroptosis microenvironment directs lineage commitment in liver cancer. Nature..

[9] Ma L, Hernandez MO, Zhao Y, Mehta M, Tran B, Kelly M (2019). Tumor Cell Biodiversity Drives Microenvironmental Reprogramming in Liver Cancer. Cancer Cell.

[10] Pickup MW, Mouw JK, Weaver VM (2014). The extracellular matrix modulates the hallmarks of cancer. EMBO Rep.

[11] Shen Y, Wang X, Lu J, Salfenmoser M, Wirsik NM, Schleussner N (2020). Reduction of Liver Metastasis Stiffness Improves Response to Bevacizumab in Metastatic Colorectal Cancer. Cancer Cell.

[12] Patel SH, Camargo FD, Yimlamai D (2017). Hippo Signaling in the Liver Regulates Organ Size, Cell Fate, and Carcinogenesis. Gastroenterology..

[13] Zhang S, Zhou D (2019). Role of the transcriptional coactivators YAP/TAZ in liver cancer. Curr Opin Cell Biol.

[14] Zhao B, Li L, Lei Q, Guan KL (2010). The Hippo-YAP pathway in organ size control and tumorigenesis: an updated version. Genes Dev.

[15] Jiao S, Li CC, Hao Q, Miao HF, Zhang L, Li L (2017). VGLL4 targets a TCF4-TEAD4 complex to coregulate Wnt and Hippo signalling in colorectal cancer. Nat Commun.

[16] Jiao S, Guan JM, Chen M, Wang WJ, Li CC, Wang YG (2018). Targeting IRF3 as a YAP agonist therapy against gastric cancer. J Exp Med.

[17] Moroishi T, Hansen CG, Guan KL (2015). The emerging roles of YAP and TAZ in cancer. Nat Rev Cancer.

[18] Yimlamai D, Christodoulou C, Galli GG, Yanger K, Pepe-Mooney B, Gurung B (2014). Hippo pathway activity influences liver cell fate. Cell..

[19] Jiang J, Ye F, Yang X, Zong C, Gao L, Yang Y (2017). Peri-tumor associated fibroblasts promote intrahepatic metastasis of hepatocellular carcinoma by recruiting cancer stem cells. Cancer Lett.

[20] Herrera M, Islam AB, Herrera A, Martin P, Garcia V, Silva J (2013). Functional heterogeneity of cancer-associated fibroblasts from human colon tumors shows specific prognostic gene expression signature. Clin Cancer Res.

[21] An L, Dong C, Li J, Chen J, Yuan J, Huang J (2018). RNF169 limits 53BP1 deposition at DSBs to stimulate single-strand annealing repair. Proc Natl Acad Sci USA.

[22] Wal M, Pugh BF (2012). Genome-wide mapping of nucleosome positions in yeast using high-resolution MNase ChIP-Seq. Methods Enzymol.

[23] Zhang L, He X, Liu X, Zhang F, Huang LF, Potter AS (2019). Single-Cell Transcriptomics in Medulloblastoma Reveals Tumor-Initiating Progenitors and Oncogenic Cascades during Tumorigenesis and Relapse. Cancer Cell.

[24] Sharma A, Seow JJW, Dutertre CA, Pai R, Bleriot C, Mishra A (2020). Onco-fetal Reprogramming of Endothelial Cells Drives Immunosuppressive Macrophages in Hepatocellular Carcinoma. Cell..

[25] Liu S, Li N, Yu X, Xiao X, Cheng K, Hu J (2013). Expression of intercellular adhesion molecule 1 by hepatocellular carcinoma stem cells and circulating tumor cells. Gastroenterology..

[26] Moreno CS (2020). SOX4: The unappreciated oncogene. Semin Cancer Biol.

[27] Wang Q, Jiang J, Ying G, Xie XQ, Zhang X, Xu W (2018). Tamoxifen enhances stemness and promotes metastasis of ERalpha36(+) breast cancer by upregulating ALDH1A1 in cancer cells. Cell Res.

[28] Shen HT, Chien PJ, Chen SH, Sheu GT, Jan MS, Wang BY (2020). BMI1-Mediated Pemetrexed Resistance in Non-Small Cell Lung Cancer Cells Is Associated with Increased SP1 Activation and Cancer Stemness. Cancers.

[29] Halder G, Dupont S, Piccolo S (2012). Transduction of mechanical and cytoskeletal cues by YAP and TAZ. Nat Rev Mol Cell Bio.

[30] Weiler SME, Lutz T, Bissinger M, Sticht C, Knaub M, Gretz N (2020). TAZ target gene ITGAV regulates invasion and feeds back positively on YAP and TAZ in liver cancer cells. Cancer Lett.

[31] Rozengurt E, Sinnett-Smith J, Eibl G (2018). Yes-associated protein (YAP) in pancreatic cancer: at the epicenter of a targetable signaling network associated with patient survival. Signal Transduct Tar.

[32] Soderstrom M, Bohling T, Ekfors T, Nelimarkka L, Aro HT, Vuorio E (2002). Molecular profiling of human chondrosarcomas for matrix production and cancer markers. Int J Cancer.

[33] Yang L, Inokuchi S, Roh YS, Song J, Loomba R, Park EJ (2013). Transforming growth factor-beta signaling in hepatocytes promotes hepatic fibrosis and carcinogenesis in mice with hepatocyte-specific deletion of TAK1. Gastroenterology.

[34] Risom T, Wang X, Liang J, Zhang X, Pelz C, Campbell LG (2020). Deregulating MYC in a model of HER2+ breast cancer mimics human intertumoral heterogeneity. J Clin Invest.

[35] Murakami S, Nemazanyy I, White SM, Chen H, Nguyen CDK, Graham GT (2019). A Yap-Myc-Sox2-p53 Regulatory Network Dictates Metabolic Homeostasis and Differentiation in Kras-Driven Pancreatic Ductal Adenocarcinomas. Dev Cell.

[36] Wang L, Zhang Z, Yu X, Huang X, Liu Z, Chai Y (2019). Unbalanced YAP-SOX9 circuit drives stemness and malignant progression in esophageal squamous cell carcinoma. Oncogene..

[37] Valkenburg KC, de Groot AE, Pienta KJ (2018). Targeting the tumour stroma to improve cancer therapy. Nat Rev Clin Oncol.

[38] Affo S, Yu LX, Schwabe RF (2017). The Role of Cancer-Associated Fibroblasts and Fibrosis in Liver Cancer. Annu Rev Pathol: Mechanisms Dis.

[39] Xiong S, Wang R, Chen Q, Luo J, Wang J, Zhao Z (2018). Cancer-associated fibroblasts promote stem cell-like properties of hepatocellular carcinoma cells through IL-6/STAT3/Notch signaling. Am J Cancer Res.

[40] Nallanthighal S, Heiserman JP, Cheon DJ (2019). The Role of the Extracellular Matrix in Cancer Stemness. Front Cell Dev Biol.

[41] Chapman A, Fernandez del Ama L, Ferguson J, Kamarashev J, Wellbrock C, Hurlstone A (2014). Heterogeneous tumor subpopulations cooperate to drive invasion. Cell Rep.

[42] Misra JR, Irvine KD (2018). The Hippo Signaling Network and Its Biological Functions. Annu Rev Genet.

