# Supplementary figures and images for: Single cell transcriptional diversity and intercellular crosstalk of human liver cancer
Source: Cell Death Dis. 2022 Mar 24;13(3):261. doi: 10.1038/s41419-022-04689-w (PMC8943132; doi:10.1038/s41419-022-04689-w)

Supplementary Material

(Western Blots)


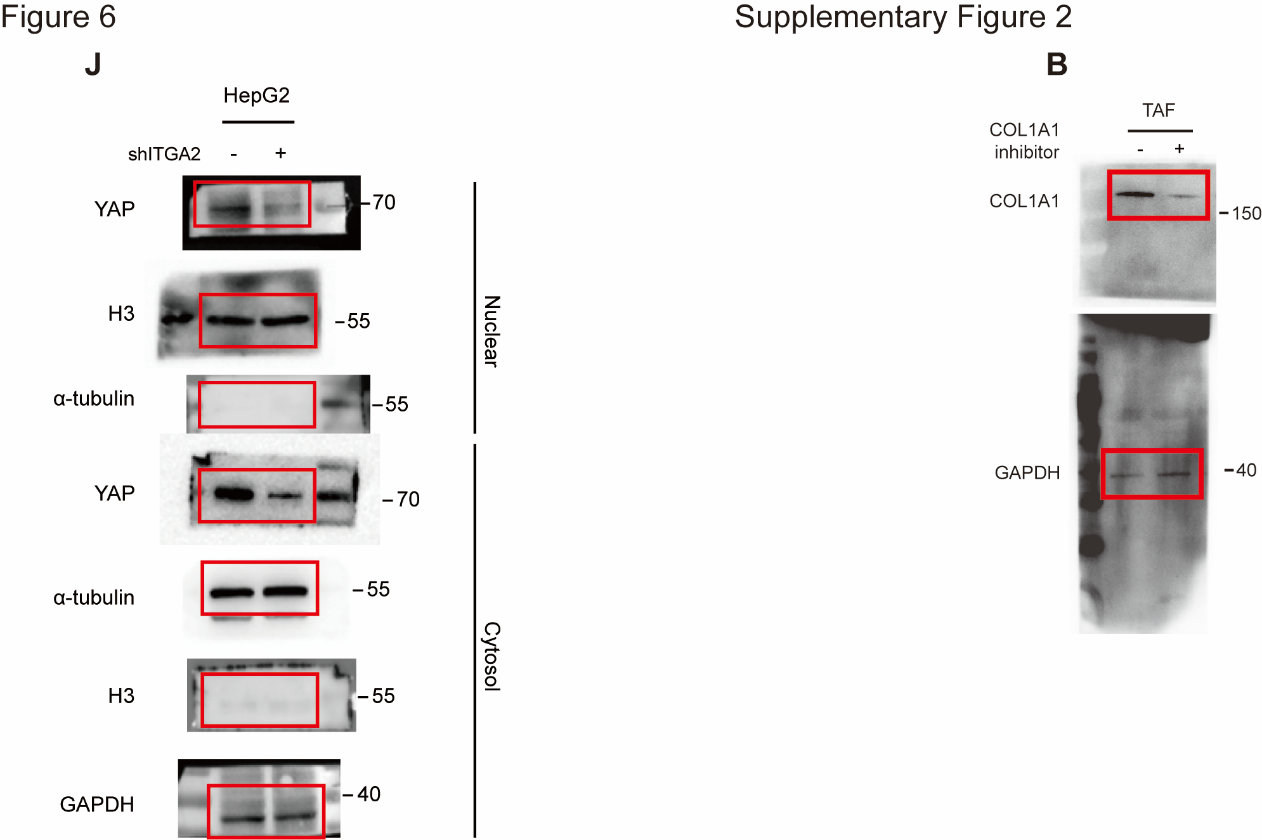

Supplement: Supplementary file 1 — Original Data File [file 41419_2022_4689_MOESM1_ESM.docx]
