# Supplementary material for: Single cell transcriptional diversity and intercellular crosstalk of human liver cancer
Source: Cell Death Dis. 2022 Mar 24;13(3):261. doi: 10.1038/s41419-022-04689-w (PMC8943132; doi:10.1038/s41419-022-04689-w)
Supplement: Supplementary file 3 — Supplemental figures [file 41419_2022_4689_MOESM3_ESM.pdf]

Supplemental Information

Single Cell Transcriptional Diversity and Intercellular Crosstalk of Human Liver Cancer

Yan Meng‡, Yan Sang‡, Jianping Liao‡, Qiudong Zhao‡, Shuping Qu, Rong Li,
Jinghua Jiang, Meifeng Wang, Jiahong Wang, Dong Wu*, Chun Cheng*, Lixin Wei*

Supplemental Figures

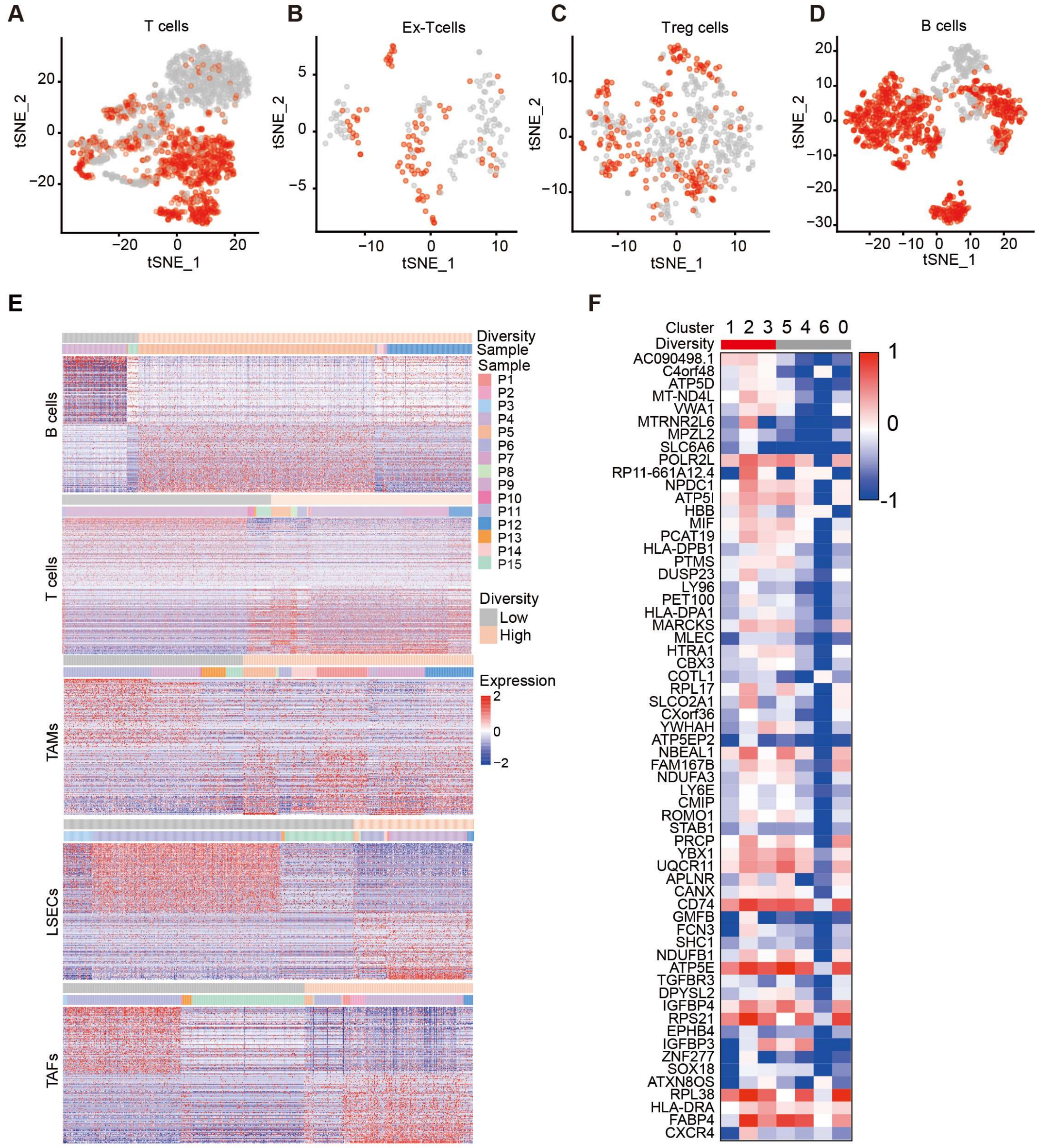

Figure S1. Analysis of Nonmalignant Cells Derived from Diversity-low and Diversity-high Group

(**A**-**D**) t-SNE plot of T cells, Exhaustive (Ex)-T cells, Treg cells and B cells from the diversity-low (gray dots) and diversity-high (red dots) groups. (**E**) Heatmap analysis of gene clusters in nonmalignant cells. The color of each group represents the level of genes. (**F**) Heatmap analysis of genes in TAMs.

Related to **Figure 2**

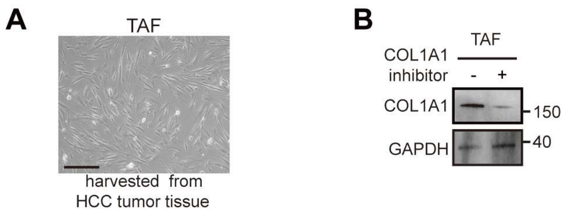

Figure S2. Establishment of Primary tumor-associated Fibroblasts (TAFs) and Silence COL1A1 in TAFs

(**A**) Microscopic examination shows the of TAFs. Bar, 100μm. (**B**) Verification of the inhibitor efficiency of COL1A1 in TAFs.

Related to **Figure 4**

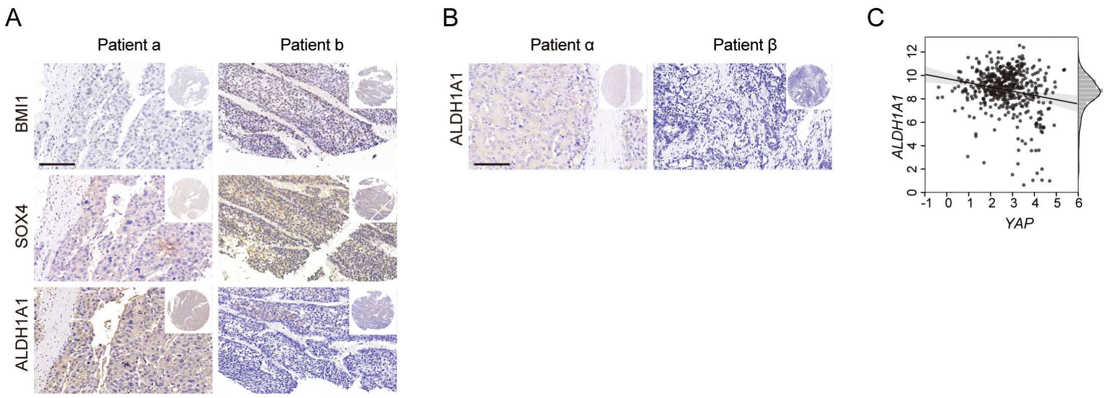

Figure S3. There was no Significant Association between the ALDH1A1and YAP

(**A)** Staining of SOX4, BMI1 and ALDH1A1 in tumor tissue from liver cancer patients. Bar, 100μm. **(B**) Staining of ALDH1A1 in tumor tissue from liver cancer patients. Bar, 100μm. (**C**) Pearson correlation analysis of the mRNA levels between ALDH1A1 and YAP in liver cancer patients from the TCGA database.

Related to **Figure 5**

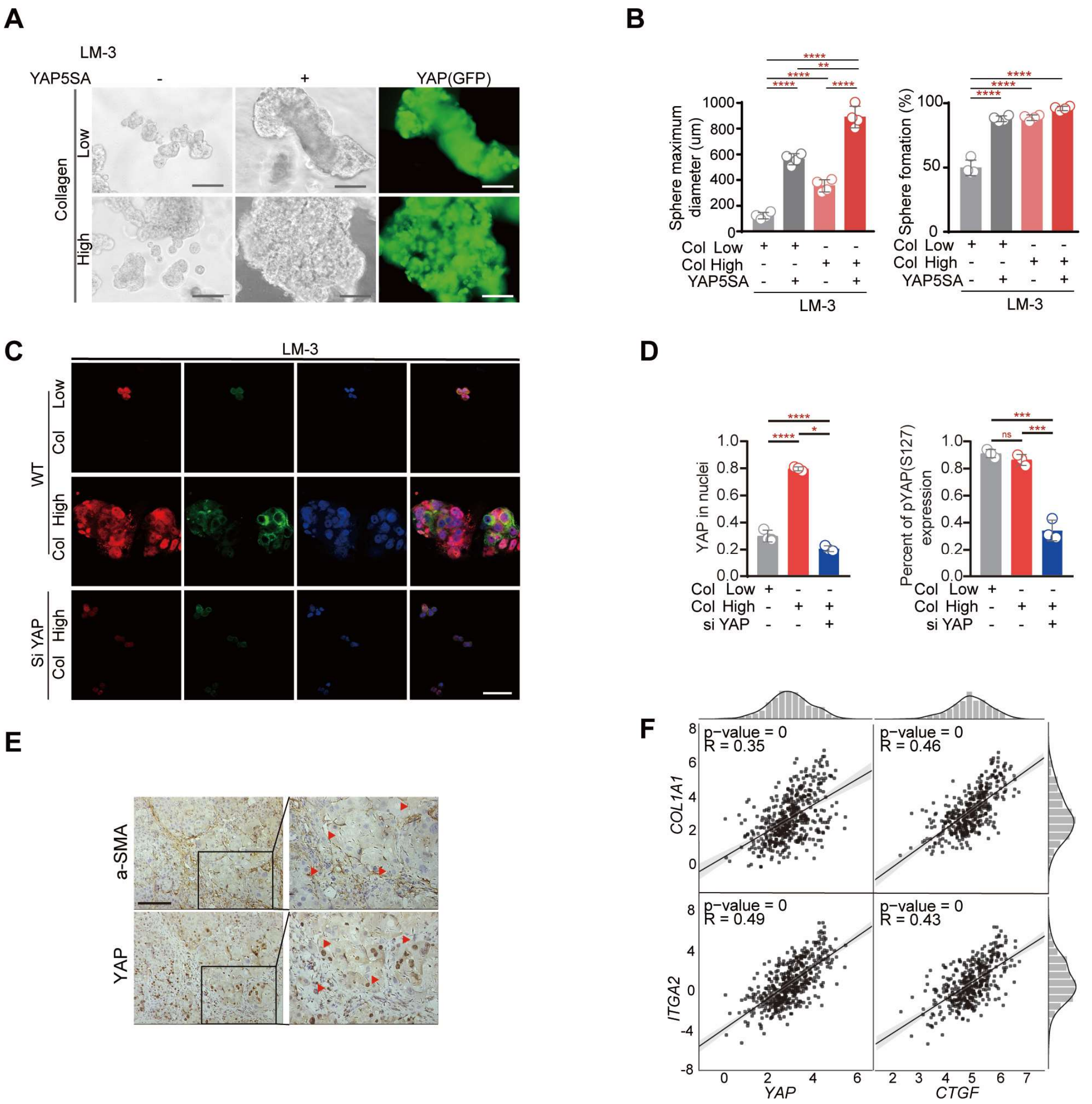

Figure S4. Collagen-mediated YAP Activation is Relate with the Stemness of Tumor Cells

(**A-B**) The capacity of colony formation was detected by sphere formation assays in LM-3 (liver cancer cell line). Cells with or without YAP overexpression (GFP-labeled) were treated with low (1 μL collagen with 3 μL DMEM) or high (collagen without DMEM) concentrations of collagen for 3 days. Bar, 200 μm. (**C-D**) The capacity of colony formation was detected by sphere formation assays in LM-3 cells. Cells with or without YAP knock-down were treated with low (1 μL collagen with 3 μL DMEM) or high (collagen without DMEM) concentrations of collagen for 3 days. Bar, 200 μm. (**E**) Staining of YAP and αSMA in tumor tissue. Bar, 100μm. (**F**) Pearson correlation analysis of the mRNA

levels of *COL1A1/ITGA2* and YAP/*CTGF* in liver cancer patients from the TCGA database. At least two independent experiments were performed for all data. Quantified data are presented as the means ± SD. Unpaired Student's t tests were used for comparing two variables and one-way ANOVA was used for comparing multiple variables. *, $P < 0.05$; **, $P < 0.01$; ***, $P < 0.001$; n.s., no significance in comparison with the control group.

Related to **Figure 6**
